# Two epitopes responsible for the catalytic activity of heme oxygenase‐1 identified by phage display

**DOI:** 10.1002/2211-5463.12217

**Published:** 2017-04-03

**Authors:** Xuran Wei, Qingjun Liu, Yaping Gao, Jun Yang, Bo Wang, Guang Yang, Shihui Zhang, Hong Zhou

**Affiliations:** ^1^ Beijing Key Laboratory of Blood Safety and Supply TechnologiesBeijing Institute of Transfusion MedicineChina; ^2^ Institute of Basic Medical SciencesAcademy of Military Medical SciencesBeijingChina

**Keywords:** catalytic activity of HO‐1, epitope, heme oxygenase‐1, phage display

## Abstract

Heme oxygenase‐1 (HO‐1) catalyzes the oxidative degradation of heme. The catalytic mechanism of the HO‐1 reaction has been determined gradually by studies of its crystal structure and HO‐1 mutants. However, the neutralizing epitopes responsible for HO‐1 activity remain elusive. Screening of a phage display library revealed four epitopes that could interact with the polyclonal antibody prepared by immunizing rabbits with the purified HO‐1 protein. Two of these four epitopes are responsible for HO‐1 catalytic activity because their antibodies were able to neutralize HO‐1 activity. The results of the present study shed further light on the molecular character of HO‐1.

**Abbreviations**
CPR: cytochrome P450 reductase
HO‐1: heme oxygenase‐1
KLH: keyhole limpet hemocyanin
rHO‐1: rat HO‐1
TBST: Tris‐buffered saline and Tween 20
ΔrHO‐1: recombinant rat HO‐1

There are two heme oxygenases, although only heme oxygenase‐1 (HO‐1) can be induced *in vivo* and is the rate‐limiting enzyme in heme degradation to CO, biliverdin and the ferrous ion (Fe^2+^) 1, 2. The high expression of HO‐1 is induced by such factors as heme, heat, heavy metals, hormone, UV, oxygen deficiency or NO 3, 4. Many disease models have shown that HO‐1 and its products (bilirubin/biliverdin, CO, Fe^2+^) have protective effects, including anti‐inflammation, anti‐apoptosis, anti‐proliferation and anti‐oxidation properties 5, 6.

Given the important biological function of HO‐1, numerous crystal structures of HO‐1 have been solved that help clarify its catalytic mechanism. The rat HO‐1 (rHO‐1) is 32 kDa in size and is composed of 289 amino acid residues. The crystal of HO‐1 with heme demonstrates that heme is sandwiched between a proximal A‐helix (Leu13‐Glu29) and a distal F‐helix (Leu129‐Met155) of HO‐1 consisting of eight α‐helices (A–H), where the His25 serves as the proximal ligand and Gly139 and Gly143 are close to the distal ligand of the heme iron 7, 8, 9. Conserved Gly143 was shown to provide the flexibility required for the opening and closure of heme activity with respect to substrate binding and product dissociation during HO‐1 catalysis 10, 11. HO‐1G143H (HO‐1 dominant‐negative mutant [Gly143 mutated to His]) could bind heme rather than transfer the electrons necessary for the catalytic reaction of HO‐1, displaying a phenotype with dominant‐negative effects. However, the neutralizing epitopes of HO‐1 remain elusive.

Phage display comprises a cheap and quick method of mapping the epitopes of the antigen involved in a specific interaction with the antibody 12. Furthermore, phage display has been used in enzymology to determine substrate specificity and to develop modulators of both the active and allosteric sites of the enzyme 13, 14, 15, 16. Epitope mapping can be performed by screening phage libraries that display random peptides encoded by either synthetic oligonucleotides or gene fragments 17, 18, 19. Thus, to identify epitopes responsible for HO‐1 activity, we used phage libraries displaying random peptides, as well as recombinant rHO‐1 (ΔrHO‐1) with high purity and activity, along with its polyclonal antibody. Two epitopes obtained by phage display screening were responsible for HO‐1 catalytic activity because their antibodies could neutralize HO‐1 catalytic activity. The findings of the present study may facilitate the identification of active sites or important functional regions of HO‐1.

## Results

### Expression and purification of ΔrHO‐1

**Figure 1:**
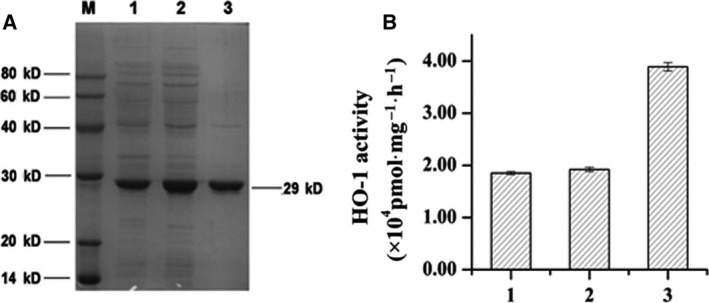
Expression and purification of ΔrHO‐1. (A) 12% SDS/PAGE analysis of purity of depuration. The protein concentrations of the samples were determined by the Brandford method. An equal amount of total proteins of samples was resolved by 12% SDS/PAGE and the purities of HO‐1 in samples were analyzed by quantity one, version 4.0 (Bio‐Rad, Hercules, CA, USA). (1) Supernatant after ultrasonication, purity 66.5%; (2) protein after salt fractionation, purity 76%; (3) protein after sieve chromatography, purity 95%. (B) catalytic activity of depurated ΔrHO‐1. (1) Supernatant after ultrasonication; (2) protein after salt fractionation; (3) protein after sieve chromatography. It is evident that the enzymatic activity was retained through purification.

Heme oxygenase‐1 is a transmembrane protein, which makes it difficult to obtain prokaryotic, soluble expression of active, full‐length rat HO‐1. For this reason, the ΔrHO‐1 (recombinant rat HO‐1) protein used in the present study was modified. We removed the transmembrane domain because Lin *et al*. 20 have shown that the transmembrane domain has no effect on the activity of HO‐1 but limits the soluble expression of HO‐1. Furthermore, the prokaryotic codons coding for HO‐1 were used for efficient expression in bacteria. The optimal expression conditions were determined by testing different temperatures and rotation speeds. The optimal expression conditions for ΔrHO‐1 were 37 °C and 200 rpm for 16 h. To retain the enzymatic activity of HO‐1, we also optimized the purification procedure. We used salt fractionation and S‐200 sieve chromatography to obtain highly active protein. As a result of SDS/PAGE (Fig. 1A), 35–55% salt fractionation removed the contaminating proteins, and S‐200 sieve chromatography separated the remaining contaminating proteins from HO‐1 by molecular weight. ΔrHO‐1 was obtained with a purity of 95%, a purification fold of 1.61 and a yield rate of 34.5%. As shown in Fig. 1B, the HO‐1 activity of expressed HO‐1 protein was well maintained after purification.

### Specificity and neutralizing activity of anti‐ΔrHO‐1 polyclonal antibodies

**Figure 2:**
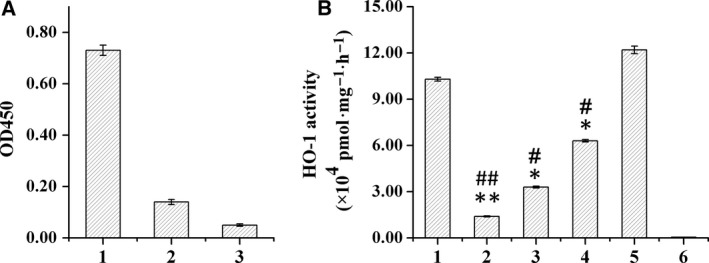
Specificity of anti‐ΔrHO‐1 polyclonal antibodies and neutralization of HO‐1 catalytic activity. (A) The specificity of anti‐ΔrHO‐1 polyclonal antibodies was detected by ELISA. Serums were diluted 1.28 × 10^7^ times. (1) and (2) Serums from two rabbits immunized with ΔrHO‐1; (3) unimmunized rabbit serum. The serum of the rabbit 1 has more binding activity (i.e. higher titer), which is why it was used in the subsequent test of the neutralizing activity of epitopes and the screening. (B) Result of neutralization of HO‐1 catalytic activity by anti‐ΔrHO‐1 polyclonal antibody. (1) Normal rabbit serum; (2) anti‐ΔrHO‐1 rabbit serum; (3) anti‐ΔrHO‐1 rabbit serum diluted 10^6^ times; (4) anti‐ΔrHO‐1 serum diluted 10^12^ times; (5) no serum in the reaction system as positive control; (6) no NADPH in the reaction system as negative control. Data are the mean ± SD (*n* = 4). **P < 0.01 compared to positive control; *P < 0.05 compared to positive control; ##*P* < 0.01 compared to control serum; #*P* < 0.05 compared to control serum.

The titers of the anti‐ΔrHO‐1 polyclonal antibody (described in the Materials and methods) from two rabbits immunized with purified ΔrHO‐1 were more than 6.4 × 10^6^ (data not shown). The results of the ELISA (Fig. 2A) demonstrated that the anti‐ΔrHO‐1 polyclonal antibodies could specifically bind to HO‐1. The neutralizing activity of the anti‐ΔrHO‐1 polyclonal antibodies was tested (Fig. 2B) and the prepared antibodies were able to neutralize the catalytic activity of ΔrHO‐1 by 72.6%. Thus, bioactive ΔrHO‐1 and its polyclonal antibody that were able to neutralize HO‐1 catalytic activity met the requirements of the screening epitopes responsible for HO‐1 catalytic activity by phage display.

### Screening and analysis of ΔrHO‐1 epitopes

**Figure 3:**
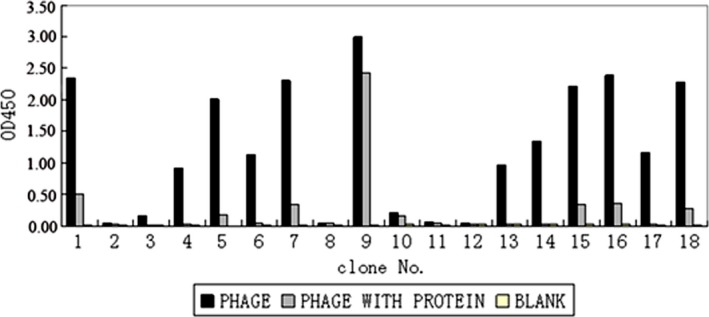
Competitive ELISA to select specific phage clones binding to anti‐ΔrHO‐1 antibodies. 96‐well plates coated with anti‐HO‐1 IgG were incubated with screened phage clones. Eighteen phage clones were selected to evaluate the binding activity to HO‐1 protein by competitive ELISA described in the Materials and methods. It can be seen that clones 1, 3, 4, 5, 6, 7, 13, 14, 15, 16, 17 and 18 might be positive clones because there were significant differences in *A*_450_ between phage and phage with HO‐1 protein. The 12 clones were used for sequencing.

**Figure 4:**
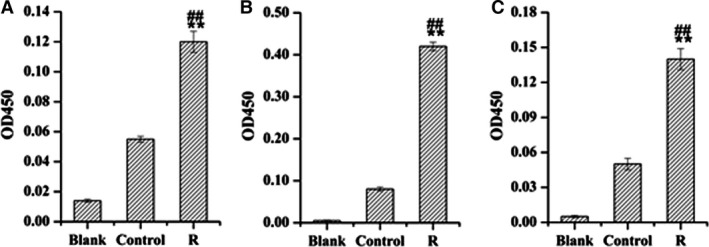
Affinity between synthetic peptides and anti‐HO‐1 antibody detected by ELISA. Plates coated with synthetic peptides [(A) A1 peptide; (B) natural peptide of A1; (C) A13 peptide] (100 μg per well) were incubated with anti‐HO‐1 antibody diluted 500 times. Blank, blank well control; Control, normal rabbit serum; R, anti‐ΔrHO‐1 serum. Data are the mean ± SD (*n* = 2). **P < 0.01 compared to blank control; ##*P* < 0.01 compared to control serum.

After three rounds of biopanning, 18 phage clones were recognized by purified anti‐ΔrHO‐1 antibody that might contain the epitopes responsible for HO‐1 catalytic activity. Twelve of these clones were selected for sequencing according to the results of the competitive ELISA (Fig. 3). The epitope sequences of the twelve positive phage clones were analyzed for four groups of peptides (Table 1). Groups A1 and A13 had more repeated clones than the other groups, suggesting that these two groups might be representative epitopes of HO‐1. Sequence analysis showed that the sequence of A1 (DYDRYSSALIAA) corresponded to its natural peptide (A_134_ YTRYLGDLSGG_144_) of HO‐1; the sequence of A3 (YWTPGYTPSQTE) corresponded to its natural peptide (E_223_EHKDQSPSQTE_234_); the sequence of A7 (NNSRDTALRWFV) corresponded to its natural peptide (T_261_SSSQTPLLRWV_272_); and the sequence of A13 (GQPKTFLSVSEL) corresponded to three natural peptides: (Y_107_TPATQHYVKRL_118_), (E_202_AKTAFLLNIEL_213_) and (S_257_ QISTSSSQTPL_268_). Because the natural peptide of A1 contained Gly143, which was a key site of HO‐1, we synthesized A1, its natural peptide and A13, and then tested these synthetic peptides by ELISA. The three synthetic peptides were able to bind to the anti‐HO‐1 antibody (Fig. 4).

**Table 1:** Sequences of specific phage clones

Group	Sequence	Repeat times
A1	DYDRYSSALIAA	5
A3	YWTPGYTPSQTE	1
A7	NNSRDTALRWFV	1
A13	GQPKTFLSVSEL	5

Repeat times indicate the number of times that the same phage clone was picked out of a total of 12 clones.

### Anti‐ΔrHO‐1 epitope antibodies and their binding activity to ΔrHO‐1

**Figure 5:**
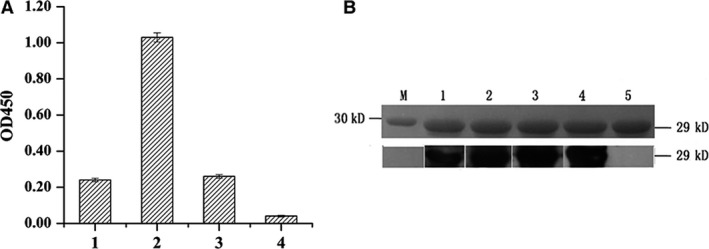
Binding activities of anti‐peptides antibodies to ΔrHO‐1 detected by ELISA and western blotting. (A) ELISA results demonstrated that the antibodies specific to epitopes screened by phage display could bind to ΔrHO‐1 protein. The antibodies were purified using DEAE‐Sephadex A‐50 (GE Healthcare) and were diluted 6400 times. (1) Anti‐A1 peptide IgG; (2) anti‐A1 natural peptide IgG; (3) anti‐A13 peptide IgG; (4) control IgG. (B) Western blotting indicated that the antibodies specific to epitopes screened by phage display could bind to ΔrHO‐1 protein. Upper tracing shows the result of SDS/PAGE; the lower tracing is the result of western blotting. The sample was purified ΔrHO‐1 protein. (1–3) Anti‐A1, anti‐natural peptide and anti‐A13 antibodies, respectively, were used as the first antibodies, which were diluted 100 times; (4) anti‐ΔrHO‐1 polyclonal antibody was used as the first antibody, diluted 5000 times, as a positive control; (5) normal rabbit serum was used as the first antibody, diluted 5000 times, as a negative control.

Purification of anti‐ΔrHO‐1 epitope antibodies prepared from rabbits immunized with synthetic peptide coupled with keyhole limpet hemocyanin (KLH) was achieved using CNBr‐activated Sepharose 4B affinity chromatography. Their titers were determined to be approximately 10^5^ (Fig. 5A). Western blotting (Fig. 5B) further showed that the purified anti‐ΔrHO‐1 epitope antibodies could specifically recognize HO‐1 protein.

### Antibodies of epitopes screened by phage display could neutralize the catalytic activity of ΔrHO‐1

**Figure 6:**
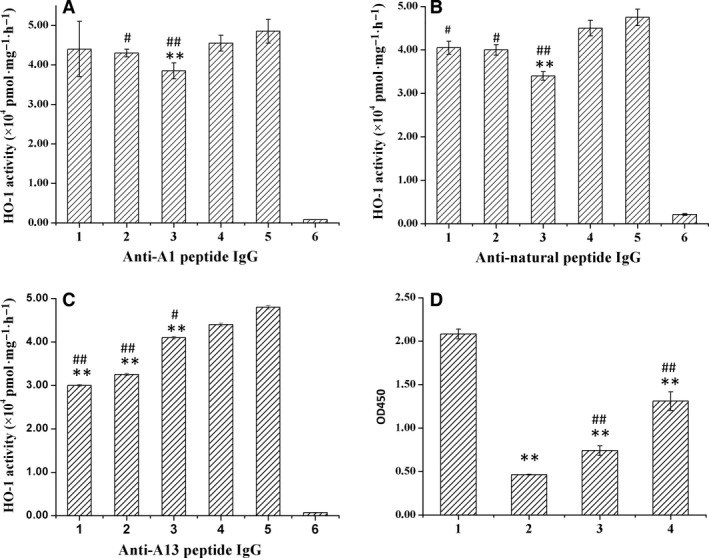
The ability of anti‐peptides antibodies to neutralize HO‐1 catalytic activity. The neutralizing catalytic activity of HO‐1 was tested by HO activity assay. (A) anti‐A1 peptide IgG. (B) anti‐A1 natural peptide IgG. (C) anti‐A13 peptide IgG. (1) Anti‐peptides IgG (1 μg·μL^−1^); (2) anti‐peptides IgG (1 × 10^−2^ μg·μL^−1^); (3) anti‐peptides IgG (1 × 10^−4^ μg·μL^−1^); (4) control IgG; (5) positive control, without IgG; (6) negative control, without NADPH. Data are the mean ± SD (*n* = 4). **P < 0.01 compared to positive control; ##*P* < 0.01 compared to control IgG; #*P* < 0.05 compared to control IgG. Data are the mean ± SD (*n* = 4). (D) Competitive experiments using anti‐A1 peptide IgG, natural peptide and purified ΔrHO‐1. (1) No natural peptide; (2) 100 μL of 0.07 μg·μL^−1^ natural peptide; (3) 100 μL of 0.035 μg·μL^−1^ natural peptide; (4) 100 μL of 0.015 μg·μL^−1^ natural peptide. **P < 0.01 compared to (1); ##*P* < 0.01 compared to (2).

The neutralizing HO‐1 catalytic activities of synthetic peptide antibodies were tested using the HO‐1 activity assay (Fig. 6). As shown in Fig. 6A, the antibody specific to A1 decreased the catalytic activity of HO‐1 when it was diluted 100‐fold. The antibody specific to the natural peptide of A1 also significantly decreased the catalytic activity of HO‐1 when it was diluted 100‐fold. Furthermore, its effect was more significant than the effect of A1 (Fig. 6B). However, the effects of the neutralizing HO‐1 catalytic activity of anti‐A1 peptide IgG and anti‐A1 natural peptide were small and there was no apparent dose dependence. The results of competitive experiment showed that the natural peptide of A1 could competitively bind to anti‐A1 peptide IgG and then block the binding of anti‐A1 peptide IgG to rat HO‐1 protein (Fig. 6D), suggesting that anti‐A1 peptide IgG might bind around the Ala134‐Gly144 of rat HO‐1. Figure 6C shows that the antibody specific to A13 peptide neutralized the ΔrHO‐1 catalytic activity by 38.7% (Fig. 6C) and the neutralizing effect decreased in a dose‐dependent manner. Thus, we concluded that both A1 and A13 are the epitopes responsible for HO‐1 catalytic activity and also that their antibodies play different inhibiting roles in HO‐1 catalytic activity.

## Discussion

Many detailed biochemical studies have been carried out with HO‐1, which is a 32 kDa protein anchored to the microsomal membrane through a C‐terminal hydrophobic tail 21. A soluble active fragment can be released from the membrane by limited proteolysis 22. The development of high‐level bacterial expression systems for a soluble form of HO‐1 missing the last 23–26 amino acids 23, 24 has led to significant advances in the study of the HO mechanism and its crystal structure, including the crystal structure of rat HO‐1 25. In the present study, we show that two epitopes of ΔrHO‐1 screened by phage display were responsible for HO‐1 catalytic activity because their antibodies could neutralize HO‐1 catalytic activity.

During the phage display screening for epitopes, the efficiency and results were affected by a number of factors, such as the titer of the target molecule (antibody), as well as the purity and the composition of the wash buffer, elution buffer and blocking buffer. Anti‐ΔrHO‐1 polyclonal antibody with a purity exceeding 90% laid the foundation for successfully screening epitopes by phage display. Furthermore, with each elutriation, the amount of antibodies used and the nonspecific binding decreased, thus accelerating the screening process. The addition of normal animal IgG into the Tris‐buffered saline and Tween 20 (TBST) used for wash buffer, the addition of 5% BSA into the blocking buffer and the addition of 0.5–1% BSA into the wash buffer also decreased nonspecific binding.

Two epitopes (A1 and A13) of ΔrHO‐1 were screened out by phage display and sequence analysis. The amino acid sequence of A1 corresponded to that of its natural peptide, indicating that A1 might be a linear epitope. The antibody of A1 had the same effect on the neutralization of the catalytic activity of HO‐1 as the antibody of the natural peptide of A1, suggesting that the antibody covering Gly143 of HO‐1 could neutralize HO‐1 catalytic activity, as confirmed in previous studies 7, 10, 11. However, it was not known why the antibodies of A1 and it natural peptide could neutralize HO‐1 catalytic activity more effectively when they were diluted to 10^4^ times. Unlike A1, the amino acid sequence of A13 corresponded to three epitopes in HO‐1, indicating that the antibody of A13 was binding to HO‐1 protein differently from the antibody of A1. However, western blotting showed that the antibody of A13 could bind to denatured HO‐1 protein, demonstrating that A13 did not appear to be a conformational epitope. Thus, the antibody of A13 might neutralize HO‐1 catalytic activity by binding to three epitopes of HO‐1. Furthermore, the antibody of A13 could neutralize HO‐1 catalytic activity in a dose‐dependent manner. HO‐1 activity is inhibited by caveolin‐1 (82–101) through the binding motif of rat HO‐1 (F_207_LLNIELF_214_) in a competitive manner with hemin. F_207_ and F_214_ play important roles in their interaction because the affinity between HO‐1 and caveolin‐1 (82–101) was almost completely or remarkably eliminated by replacement of F_207_ and/or F_214_ with Ala 26. One of the natural peptides corresponding to A13 (E_202_AKTAF207LLNIEL_213_) includes F_207_ and is adjacent to F_214_; thus, the way in which the HO‐1 catalytic activity of the antibody of A13 is neutralized may be the same as that of caveolin‐1.

In summary, two epitopes responsible for the catalytic activity of HO‐1 were screened out by phage display, which might shed further light on the biological character of HO‐1, as well as future manipulations specific to HO‐1.

## Materials and methods

### Expression and purification of ΔrHO‐1

To express rHO‐1 efficiently in bacteria, we used the *ΔrHO‐1* DNA sequence with prokaryotic codons and deletion of membrane anchor region coding the 22 C‐terminal residues. The sequence synthesized by Biomed (Beijing, China) was inserted into the *Nde*I and *Hin*dIII sites of the pMW172a plasmid, which contains the phage T7 promoter. The pMW172/ΔrHO‐1 plasmid was transformed into *Escherichia coli* BL21 (DE3) cells to express the ΔrHO‐1 protein, whereas the empty vector pMW172 was used as a control. To determine the optional expression condition, we compared the expression of ΔrHO‐1 at 37 °C and 200 rpm for 16 h, at 37 °C and 200 rpm and 0.1 mmol·L^−1^ isopropyl thio‐β‐d‐galactoside for 16 h, and at 37 °C and 120 rpm for 16 h. Then, the ultrasonicated bacteria expressing ΔrHO‐1 were centrifuged at 18 000 ***g*** for 15 min at 4 °C. The ΔrHO‐1 protein was precipitated, respectively, from the supernatant at concentrations of 5–15%, 15–25%, 25–35%, 35–45%, 45–55% and 55–65% of (NH4)_2_SO4 at 4 °C for 3 h before the dissolved precipitation with PBS was graded using Sephacryl S‐200HR sieve chromatography (GE Healthcare, Milwaukee, WI, USA) at *A*_280_ by means of AKTA FPLC (Amersham Pharmacia, Piscataway, NJ, USA). The effects of purification for ΔrHO‐1 protein were analyzed by 12% SDS/PAGE.

### Production, purification and titer of polyclonal anti‐ΔrHO‐1 antibodies

The antibody was prepared by immunizing New Zealand rabbits with purified ΔrHO‐1 three times by hypo‐multi drop injection, once every 2 weeks. Emulsive HO‐1 with complete Freund's adjuvant was used in the initial immunization, whereas emulsive HO‐1 with incomplete Freund's adjuvant was used in subsequent immunizations. Then, femoral artery cannulation was used to collect blood that was centrifuged at 966 ***g*** for 15 min to separate the serum and stored at −80 °C. The anti‐ΔrHO‐1 polyclonal antibody was purified by affinity chromatography using CNBr‐activated Sepharose 4B (GE Healthcare) coupled to ΔrHO‐1 and DEAE‐Sephadex A‐50 (GE Healthcare) in accordance with the manufacturer's instructions.

The titer of antibody produced by rabbits was determined by indirect ELISA. Briefly, purified rHO‐1 protein was coated in 96‐well plates (Nunc, Rochester, NY, USA) (100 μg per well) followed by incubation overnight at 4 °C. The plates were then washed with washing buffer (PBS with 0.05% Tween 20) five times for 3 min before 100 L of diluted anti‐serum was added followed by incubation at 37 °C for 1 h. After being washed four times, HRP‐conjugated anti‐rabbit IgG (dilution 1 : 5000) was added followed by incubation at 37 °C for 0.5 h. After being washed, 100 μL of TMB (3,3′,5,5′‐tetramethyl benzidine dihydrochloride) substrate (Sigma, St. Louis, MO, USA) was added for 10 min and terminated by stop buffer. The color intensity was determined spectrophotometrically at *A*_450_. The titer of the serum is the diluted times of serum when the OD450 ratio of diluted serum and control serum is 2.1.

### HO‐1 activity test

To test HO‐1 activity, a reaction system was used in which cytochrome P450 reductases (CPR) supply electrons and comprised 25 μmol·L^−1^ hemin, 5 μmol·L^−1^ biliverdin reductase (BRE), 5 μmol·L^−1^ CPR, 1 mmol·L^−1^ NADPH and 5 mmol·L^−1^ deferoxamine mesylate. The reaction was incubated for 1 h at 37 °C in the dark and terminated by the addition of 0.5 mL of chloroform. The amount of extracted bilirubin was calculated as the difference in absorption between 464 and 530 nm using a quartz cuvette 4. HO activity was calculated as 31 250 × Δ*A*/Cp (picomol bilirubin mg protein^−1^·h^−1^). Cp was the concentration of HO‐1 protein (mg·mL^−1^).

### Phage library and screening

Selection was carried out in accordance with the protocol of the PhD™ phage display peptide library kit (New England Biolabs, Beverly, MA, USA). In brief, purified anti‐ΔrHO‐1 polyclonal antibodies were coated to 96‐well plates (Nunc) (10 μg per well) and then 4 × 10^10^ phages were added for incubation at 4 °C for 1 h. After being washed six times with 0.1% TBST, the binding phages were eluted to infect *E. coli* ER2537. The level of specific phage enrichment was calculated as the ratio of the input to the output as described in the manual 27. After three rounds of biopanning, 10 μL of phages mixed with 200 μL of *E. coli* ER2537 was placed on the LB agrose plate.

### Positive phage clones screened by competitive ELISA and sequencing

The selection of positive phage clones specific to polyclonal anti‐ΔrHO‐1 antibodies was detected by competitive ELISA. Briefly, polyclonal antibodies were applied to 96‐well plates (Nunc) (10 μg per well) followed by incubation overnight at 4 °C. Unbound anti‐ΔrHO‐1 antibodies were removed, and the wells were blocked with 3% BSA in PBS at 37 °C for 1 h. Eighteen selected phage clones (1 × 10^9^ per well) and diluted purified ΔrHO‐1 were added, respectively, followed by incubation at 37 °C for 2 h. The plates were then washed with washing buffer (PBS with 0.05% Tween 20) five times for 3 min, and an anti‐phage M13 monoclonal antibody (dilution 1 : 1000) (Amersham Pharmacia) was added followed by incubation at 37 °C for 1 h. After washing as described above, the binding of anti‐phage antibodies was detected using TMB, and the color intensity was determined spectrophotometrically at *A*_450_. Twelve phage clones were sequenced from the eighteen ones with M13‐96 primer to determine the amino acid sequences of epitopes.

### Western blotting for specificity of antibodies

The specificity of the antibodies was detected by western blotting. A 10 μL aliquot of each protein sample was subjected to 12% SDS/PAGE and then proteins were electrophoretically transferred to a poly(vinylidene difluoride) membrane. Blotting was performed with a current of 200 mA for 1 h at 4 °C using transfer buffer consisting of 0.025 m Tris base, 0.192 m glycine and 20% methanol (pH 8.3). The blots were blocked overnight at 4 °C in blocking buffer [TBS (pH 7.4), 0.1% Tween 20 and 5% nonfat dried milk], incubated for 1 h at room temperature with the prepared anti‐HO‐1 polyclonal antibodies diluted to 1 : 5000 with blocking buffer, washed three times with TBST (TBS with 0.1% Tween 20), incubated for 0.5 h at room temperature with anti‐rabbit IgG‐horseradish peroxidase diluted to 1 : 5000 with blocking buffer, and then washed six times with TBST. The transferred proteins were incubated with an ECL substrate solution for 3 min in accordance with the manufacturer's instructions (Amersham Pharmacia) followed by visualization with X‐ray film (Kodak, Tokyo, Japan).

### Peptide synthesis

The peptides were synthesized by the Institute of Basic Medical Sciences of the Academy of Military Medical Sciences (Beijing, PR China). The peptides coupled with KLH were used for preparation of epitope antibodies from rabbits as described in the Materials and methods.

### Neutralization of HO‐1 catalytic activity of antibodies

The neutralizing HO‐1 catalytic activity of the antibodies was tested using the HO‐1 activity assay as described above.

### Competitive experiment of anti‐A1 peptide IgG to its natural peptide andΔrHO‐1 protein

The binding activity of anti‐A1 peptide IgG to the natural peptide of A1 and ΔrHO‐1 protein was detected by competitive ELISA. Briefly, purified ΔrHO‐1 protein was applied to 96‐well plates (Nunc) (10 μg per well) followed by incubation overnight at 4 °C. Unbound proteins were removed, and the wells were blocked with 5% BSA in PBS at 37 °C for 1 h. Next 50 μL of anti‐A1 peptide IgG was mixed with natural peptide at different concentrations at room temperature for 1 h. Then, 100 μL of the mixtures were added to the wells for 2 h at room temperature. After being washed, goat‐anti‐rabbit IgG‐HRP (dilution 1 : 2000; Santa Cruz Biotechnology, Santa Cruz, CA, USA) was added to the plate. TMB substrate was used for coloring. The color intensity was determined spectrophotometrically at *A*_450_.

## Author contributions

QL, JY, GY and HZ conceived and designed the experiments. XW, QL, YG, BW and SZ performed the experiments. QL analyzed the data and wrote the paper.
